# Decision-making on terminating pregnancy for Muslim Arab women pregnant with fetuses with congenital anomalies: maternal affect and doctor-patient communication

**DOI:** 10.1186/s12978-017-0312-7

**Published:** 2017-04-04

**Authors:** Anat Gesser-Edelsburg, Nour Abed Elhadi Shahbari

**Affiliations:** School of Public Health, University of Haifa, 199 Aba Khoushy Ave. Mount Carmel, Haifa, 3498838 Israel

**Keywords:** Decision-making on terminating pregnancy, Pregnant Muslim Arab women, Fetuses with congenital anomalies, Maternal affect, Doctor-patient risk communication

## Abstract

**Background:**

This study focused on decision-making on terminating pregnancy for Arab Muslim women in Israel who were pregnant with fetuses diagnosed with congenital anomalies. It examined the impact of the doctor-patient interaction on the women’s decision, especially in light of social and religious pressures not to terminate under any circumstances. Our goal was to identify perceptions and attitudes of Muslim Arab women who choose to continue their pregnancy following the detection of congenital anomalies in prenatal tests. Specific objectives included (1) To examine the Muslim Arab women’s perceptions on genetic testing, and ascertain the reasons for their decision to continue the pregnancy following the detection of a congenital anomaly in the fetus; and (2) To examine risk communication of gynecologists regarding genetic testing and abortions, and regarding the decision of continuing or terminating a pregnancy following detection of a congenital anomaly.

**Methods:**

The research framework used the constructivist classical qualitative method to understand the experience of women at high risk for congenital anomalies and their experience of how doctors communicate the risk.

**Results:**

It showed that the emotional element is no less dominant than religious and social elements. The findings emphasized the disparities between doctors and women regarding emotional involvement (non-directive counselling). The women interviewees (*N* = 24) felt that this expressed insensitivity. As far as we know, the emotional component has not been raised in previous studies of Muslim women at high risk for congenital defects in their fetus, and therefore comprises a significant contribution of the present study.

**Conclusions:**

To mitigate gaps, doctors should take affect into consideration in their communication with patients. It is important for doctors to understand the emotional element in risk communication, both in how they respect women’s emotions and in creating an emotional interaction between themselves and the women.

## Plain English summary

This study focused on decision-making for Arab Muslim women in Israel who were pregnant with fetuses diagnosed with congenital anomalies. It examined the impact of the doctor-patient interaction on the women’s decision to continue the pregnancy, especially in light of social and religious pressures not to terminate the pregnancy under any circumstances.

It showed that the emotional element is no less dominant than religious and social elements. The women mentioned that they felt the fetus’s movements and that giving it up would be giving up motherhood. The present study also found that fatalism is also a factor in choosing to continue the pregnancy. The findings emphasized the disparities between doctors and women regarding emotional involvement (non-directive counselling). The women interviewees felt that this expressed insensitivity. This has not been raised in previous studies of Muslim women at high risk of giving birth to a fetus with a defect, and that is a significant contribution of the present study.

We recommend that doctors consider the emotional element in risk communication, or to arrange for a social worker to accompany them in their meetings with patients to offer emotional support to female patients who face a difficult decision.

## Background

### Congenital anomalies in the fetus

The detection of congenital anomalies in fetuses is an important component of prenatal health services and usually entails a decision to continue or terminate the pregnancy [1]. Deciding whether to terminate a pregnancy after the detection of a congenital anomaly is dependent on multiple factors: maternal age, reproductive history, religious and cultural beliefs, stances on terminating pregnancy, ultrasound imagery of the anomaly, certainty of the diagnosis, stage of pregnancy, severity of the defect, number of organs affected, and specific organ systems affected [2]. The literature indicates that traditional populations are more inclined than modern populations to decline genetic testing [3] and pregnancy termination [4]. Congenital anomalies are also known as birth defects, congenital disorders or congenital malformations. Congenital anomalies can be defined as structural or functional anomalies (for example, metabolic disorders) that occur during intrauterine life and can be identified prenatally, at birth, or sometimes may only be detected later in infancy, such as hearing defects. In simple terms, congenital refers to their existence at or before birth [5].

This study examined a subpopulation of Muslim Arab women in Israel with high risk pregnancies whose fetuses were diagnosed with congenital anomaliesand who decided against terminating pregnancy. Despite the high risk for congenital anomalies in this subpopulation relative to other subgroups in Israel [6], we know of no studies of this population and how they contend with the option of terminating pregnancy [7]. Moreover, the studies that have been conducted involving Muslims in Israel and Muslims in other countries tend to examine parents who already had children with congenital anomalies, or the attitudes of parents of healthy children towards genetic testing and abortion [8]. Previous studies about opting in favor of continuing the pregnancy after the detection of congenital anomalies have been conducted in Israel among Bedouin Arabs. Although Muslim, the Bedouin are not representative of the Israeli Muslim population for several reasons, including that most do not have community clinics for the provision of either preventive or curative health services, and must travel to clinics in the towns or cities for their health care [9–11]. Notably, many Bedouin women go to a prenatal exam only in the second trimester [12].

Muslim clergymen in Israel were not found to oppose termination during the first 120 days of pregnancy if a congenital anomaly was detected [13, 14]. Studies of Arab Muslims worldwide have found that religious reasons often prevent women both from undergoing prenatal genetic testing [15, 16] and choosing pregnancy termination [17–19].

Studies of Muslim populations in Western countries have found a wide range of positions on terminating pregnancy [20]. Some demonstrate the power of cultural and religious considerations in Muslim women’s decisions on this issue [3, 14, 21]. Other factors in the decision to continue the pregnancy are related mostly to socioeconomic status, traditional beliefs, education level, disease severity, and the burden it imposes on immediate family [22–25].

In Israel, abortion has been legal since 1977, and is covered by the national insurance under the following conditions: the woman is under age 17 or over age 40; the woman is unmarried; the pregnancy is the product of a rape or incest; the pregnancy threatens the mother’s physical or emotional health; or if the fetus was found to have congenital anomalies [26].

Among Arabs in Israel, about 16% of babies born to couples who are first cousins suffer from some kind of genetic anomaly, while if the parents are related more distantly, the rate is 15.2%. In contrast, when the parents are not related, then the rate of genetic anomaly among Arabs in Israel is 8.3% of births, and some villages have a rate as low as 5.8% of births to parents who are not related [8]. Studies that examine attitudes of Arab women in Israel showed that 35% said they would not consider terminating a pregnancy even in the event of a fetus with a genetic anomaly. Another 35% would consider terminating a pregnancy only during the first 120 days of the pregnancy. Only 22% reported that they would do so in the event of a congenital anomaly. There was no significant statistical difference in answers of those from different socio-economic, demographic or religious backgrounds. However, about 70% of the respondants said they had a relative who was born with a congenital anomaly. About 50% of the respondants who said they would not consider pregnancy termination cited religious reasons for this, while 48% cited their husband’s stance as the determining factor [27]. It is possible that the women’s personal interaction with family members who have congenital anomaliesaffects their willingness to raise children born with such anomalies, along with the high prevalence of marriage between first cousins among Israeli Arabs, which stands at about 44% [28].

### Maternal emotion

One reason that women decide to continue pregnancies at high risk for anomalies is the mother’s attachment to her fetus. In what Winnicott [29] calls “primary maternal preoccupation”, he indicates that the connection between pregnant women and their fetuses is established during the pregnancy. Raphael-Leff [30] has shown that pregnant women fantasize about their fetuses. Cohen, Slade [31] show that many women tend to project their fears, hopes, dreams and memories onto the fetus. Cranley [32] coined the term “maternal-fetal attachment” to describe the burgeoning relationship between the pregnant woman and her fetus, including behaviors and thoughts that reflect the establishment of an emotional connection.

The theory of “maternal role attainment” suggests four stages of women’s decision-making processes: anticipatory, formal, informal, and personal identity [33]. Lalor et al. [34] examined the choices of women with high risk pregnancies to undergo the maternal serum screening (MSS) test. The study claimed that pregnant women begin to bond with the fetus in early stages of pregnancy. In contrast, there are studies that revealed the difficulty of pregnant women to develop feelings towards the fetus upon diagnosis of fetal anomalies. Skotko [35] studied women who decided to continue their pregnancies despite diagnosis of Down Syndrome, and found that the woman’s “maternal ethnicity” led them not to terminate the pregnancy.

### Affect in risk communication

There are different approaches towards communication and counseling provided by doctors. The nondirectiveness approach is taken to imply, almost implicitly, moral neutrality on the part of the counsellor, with only the client’s values having any part in decision making. This must be set against research that reports mismatches between the judgments of health professionals and patients in relation to factors such as the patient’s need for information and her or his emotional state [36, 37]. Harper & Clarke [38] argues that nondirectiveness creates an “emotional distance” from clients, thus protecting the professional from over-involvement and ensures that professionals will not be held legally accountable for decisions made by clients.

In contrast with this approach, there is an approach that relates to emotion, empathy and affect. The importance of affect on decision making is recognized increasingly in the literature [39–41]. Several authors argue that people can evaluate risks and risk information using affective and cognitive appraisals [42]. Slovic et al. [43] opine that patients and doctors must often make decisions in environments with high levels of affect (at least for the patient) and high levels of vulnerability. Therefore, the doctor-patient dialog and its outcomes are informed by affect, alongside cognitive considerations [44].

### Emotions and medical care

The various stages of medical care involve communication between doctors and patients. Care communication involves communicating the risk of a congenital anomaly to the patient, and is part of risk communication. This communication is significant for the quality of care and can improve the ability to diagnose a medical problem, and also improve patient compliance [45].

Patient-centered communication is widely valued as a conceptual marker of quality care, supported by a growing literature that links communication to a host of valued outcomes [46]. Empathy is seen as an essential element of the doctor-patient relationship. Research suggests a relation between physician empathy and increased patient satisfaction, as well as reduced time and expense [47].

Paling [48] believes that “most patients’ assessment of risks is primarily determined not by facts but by emotions… the most powerful precursor for effective risk communication is for the doctor to strive to display both competence and a caring approach”. Another important element of communication is the doctor’s consideration of the patient’s cultural frame of reference [49].

The scientific literature that explores why Muslim women choose to continue pregnancies following the detection of a congenital anomaly tends to focus on religious, cultural and social aspects, neglecting maternal affect. The purpose of this study was to examine the attitudes of women with high-risk pregnancies in Israel regarding pregnancy termination. We also examined the impact of doctor-patient interaction on the women’s decision.

### Objectives

Our goal was to identify perceptions and attitudes of Muslim Arab women who choose to continue their pregnancy following the detection of congenital anomalies in genetic and other prenatal tests. Specific objectives included (1) To examine the Muslim Arab women’s perceptions on genetic testing, and ascertain the reasons for their decision to continue the pregnancy following the detection of a congenital anomaly in the fetus; and (2) To examine the risk communication of gynecologists regarding genetic testing and pregnancy termination, and regarding the decision to continue or terminate a pregnancy following detection of a congenital anomaly.

## Methods

### Study design

The present research framework used the constructivist classical qualitative method research method in order to understand the experience of Muslim women at high risk for congenital anomaliesand their experience of how doctors communicate the risk [50]. The study’s theoretical model referred to affect-inducing risk communication [43].

### Study participants

The study included 29 participants: 24 patients and 5 doctors (OB/GYN). All of the women were Muslim women from northern Israel pregnant with fetuses diagnosed with congenital anomalies. All of the doctors were Arab and the communication took place in Arabic. The purpose was to examine the impact of the perceptions of family and environment. The research population included 5 doctors (OB/GYN) (Table 1) to examine risk communication between them and pregnant women (Table 2). As Table 2 shows, the patients come from a range of provenances in northern Israel – from Nazareth (city) and from nearby villages. Their socio-economic status was middle-high and therefore were able to pay for genetic testing.

**Table 1:** Doctors’ (OB/GYN) age, clinic and interview locations

Interviewee No.	Age (Years)	Clinic location	Interview location	Interviewee position
1	43	Nazareth	Community clinic	Pediatric, obstetrics and infertility expert, Director of Risk Management Department, French Hospital
2	53	Afula	Haemeq Hospital	Adminstrator of Gynecology Departmaent, Haemeq Hospital, Head of Obstetrics Department Committee of the Termination of Pregnancy, member
3	63	Nazareth	French Hospital	Administrator of the Department of Gynecology in French Hospital, infertility expert
4	42	Afula	Haemeq Hospital	Adminstrator of nursery and maternity departmaent in Haemeq hospital (head of delivery rooms), Committee of the Termination of Pregnancy, member
5	45	Nazareth	Community clinic	Adminstrator of Obstetrics Departmaent, French Hospital, Pediatric, obstetrics and infertility expert

**Table 2:** Arab Muslim women pregnant with fetuses diagnosed with congenital abnormalities (age, marital status, number of pregnancy, geographic location of interview, socio-economic status)

Interviewee No.	Age (Years)	Marital Status	Number of Pregnancy	Geographic location of Interview	Socio-Economic Status
1	31	Married	3	Nazareth (city)	Medium
2	33	Married	3	Nazareth (city)	Medium
3	21	Married	1	Nazareth (city)	High
4	24	Married	1	Daburiyya (village)	Medium
5	37	Married	4	Yafia (village)	Medium
6	25	Married	2	Nazareth (city)	Medium
7	32	Married	4	Mashhad (village)	Medium
8	27	Married	4	Reina (village)	Medium
9	32	Married	1	Sullam (village)	High
10	23	Married	1	Ein Mahl (village)	Medium
11	39	Married	4	Nazareth (city)	High
12	28	Married	3	Tayba Elzoabia (village)	High
13	23	Married	1	Nazareth (city)	High
14	32	Married	3	Kfar Manda (village)	Medium
15	31	Married	2	Nazareth (city)	High
16	23	Married	1	Daburiyya (village)	High
17	33	Married	3	Shefaram (village)	High
18	35	Married	1	Nazareth (city)	High
19	36	Married	5	Nazareth (city)	Medium
20	31	Married	3	Nazareth (city)	High
21	37	Married	3	Nazareth	High
22	38	Married	4	Kafr Kanna	Low
23	35	Married	3	Tayba Elzoabia	Medium
24	22	Married	1	Iksal	High

### Recruitment and sampling

The research took place during the years 2014–2015. The research sampling consisted of a homogeneous sample of pregnant women with positive genetic diagnosis of congenital anomalies [51]. The emphasis in a study of this kind is to select the informative group that best represents the population from which it was chosen and that can teach us about the issue under study [50]. Five interviewees were recruited by gynecologists, two by a social worker. The rest were recruited through other interviewees (snowball sampling). Most of them initiated contact with the researcher, neutralizing the risk that women would agree to be interviewed only out of a sense of obligation towards the doctor/clinic.

The doctors (OB/GYN) who participated were recruited by intensity sampling of doctors (OB/GYN), high-risk pregnancy hospital departments, and from lists of gynecologists we found on websites on gynecology, obstetrics and fertility.

### Research tools

In this study three protocols were created for the target subpopulations: the first for gynecologists, the second for women pregnant for the first time whose fetuses were diagnosed with congenital anomalies, and the third for women in their second (or later) pregnancies, but for whom it was the first time that their fetuses were diagnosed with congenital anomalies.

Interview protocols for the women included questions about life perceptions, attitudes towards genetic testing and terminating pregnancy, beliefs and norms, influence of emotions on their decision, their interaction with the attending physician who communicated the anomaly, and ascertainment of their preferred channels of communication. The protocol for women in their second pregnancies included additional questions about views on the integration of a disabled child into the family.

The protocol for the interviews with the gynecologists included questions about their communication with women pregnant with fetuses with genetic defects, their attitudes towards genetic testing and abortion, and the gynecologists’ communication channels for conveying the desirable message.

### The research process

After receiving the approval of the ethics committee at Haifa University, we approached gynecologists in Nazareth and in high-risk pregnancy wards in northern Israel. The interviewees we invited agreed to partcipate and all signed informed consent forms to confirm participation. The protocols were translated into Arabic, the language of the research population, and then back from Arabic into Hebrew to check accuracy.

The proportion of interviewees (24) was one quarter of the in-patients in the high risk pregnancy wards within the two hospitals during the 6-months period in which the study was conducted. The interviewer stopped interviewing additional women once we arrived at data saturation of the themes delineated [52]. All the women who participated were sent an explanation and a consent form and invited to a face-to-face interview to explore their experiences of high-risk pregnancy.

The purpose of the study and its importance were explained, and after receiving their agreement in principle to participate, we scheduled 50–60 min interviews (at time and place of their choice).

The questions were open-ended and probes were used to elicit more information. Recordings of the interviews were transcribed verbatim and de-identified. The Arabic-language interviews were transcribed by a researcher fluent in Arabic and Hebrew. All interviews were conducted by a researcher who had postgraduate training in qualitative research. Another reliable control was that the transcripts were translated into Hebrew, so that two researchers ascertained the findings. The research participants represent different subpopulations (women with high-risk first pregnancies, women with high-risk pregnancies with children, gynecologists), which strengthens the credibility and validity of the findings.

### Data analysis

The analysis was conducted throughout the data collection process and focused on issues as they relate to each of the specific research questions [53]. The process of analysis included open coding, creating categories and abstraction, moving from specific to more general to create a picture of the larger whole. This method of analysis was chosen to facilitate the identification of themes in the decision-making process related to pregnancy termination. We conducted inductive detection of the primary themes that emerged, using textual analysis of overt discourse to determine themes explicitly stated, and of the latent discourse [54]. Two coders read and reread transcripts separately (one in Hebrew, one in Arabic) to identify potential codes, convened to create a common coding system through discussion, and then separately coded text [55]. This process continued until agreement and matching. Qualitative data are presented through participants’ direct quotes to illustrate findings.

The analysis emphasized the component of affect both in the process of risk communication between the women and the physicians and its role in the women’s decision to continue the pregnancy. A thematic analysis was carried out within and across groups, enabling us to identify issues shared by women and doctors [56, 57].

## Results

Different topics arose regarding the women’s decision-making process, including their views on religious leaders; the husband’s degree of support; their view on the consequences of having a baby with congenital defects, etc. The same issues were not raised by every interviewee, and their reactions were not homogenous (see Table 3). All of the women raised two components: religious and emotional. In the findings, we present how the women and the doctors express the religious component. We will refer to the emotional component of the communication between the doctor and the women.

**Table 3:** Table of Considerations Affecting Abortion Refusal

Against Abortion	In favor of Abortion	Various Considerations
Interviewee #9: “I am already used to this… because my third daughter had the same illness. Medicine is extremely advanced. I know what the illness is like and how to treat it.”	Interviewee #1: “Yes, of course… there will be a pronounced difference in my lifestyle, and there will be a big difference in my family’s life and my husband’s life”	Changes in Lifestyle
Interviewee #15: “No, definitely not, with all of the advances in the world, with all of the advances and the rights that special needs are entitled to, the situation is still tragic in our society. The child’s parents will always be given looks of pity, and they will always be treated as though the child is a burden for them and for the family.	Interviewee #8: “Yes… if the child has supportive parents who help him, and if his parents teach him that he is no different from other children, and they educate him the right way, then these children can fulfil themselves in life…”	Wellbeing of a Child with Disability
Interviewee #28: “If they were to tell me that the pregnancy is endangering my life, I would not terminate the pregnancy… When it’s your time to die, it’s your time… Everything is in God’s hands… He is the one who decided when a person dies.”	Interviewee #9: “I would choose me because I have children and a sick daughter at home who really really needs me… According to our religion, it says in the Quran that if there is any danger, the woman is allowed to abort.”	Will the pregnancy put the women’s life in danger?
Interviewee #17: “My husband’s family and my husband encouraged me to abort… You know why? The whole family is secular. But their views don’t interest me at all, and don’t have any effect on me.”	Interviewee #20: “I was afraid of God. From God’s punishment if I killed a soul… the people around me put these ideas into my head.”	Family Influence
Interviewee #1: “I have no doubt about the results. OK! Like all mothers, I did not want my child to be born sick… I wanted to continue the pregnancy with a healthy child. But I can’t ignore reality.”	Interviewee #17: “I don’t even think about it. I can’t imagine having a retarded son. We won’t even think about it. I believe that I am carrying a healthy child. That’s why I am not planning to prepare for a child with special needs.”	Hope and Optimism
Interviewee #8: “His father and me… Of course… Who else other than us would take care of him?”	Interviewee #12: “My children’s main caretaker is me. Whether the child is sick or not… My husband does not help at home and he does not know how. I carry the responsbility alone.”	Husband’s degree of support
Interviewee #5: “I will have to give more time and energy to one child. I am afraid that this will be at the expense of my other son.”	Interviewee #13: “The first reason is that this is my first pregnancy… the first time I have gotten pregnant… the first time I will be becoming a mother.”	What Number Pregnancy?
Interviewee #3: “My second son is extremely jealous of my daughter… how would it be if he had an even younger brother. He’d go completely crazy… But there’s nothing I can do about this.”	Interviewee #8: “The thing that encouraged me was that after 3 daughters, I really wanted a son… If you don’t have a son, they look at you differently… it’s a matter of tradition to have a son who carries the family forward.”	The importance of the foetus’s sex
Interviewee #13: “Religious leaders don’t have anything to do with this topic. We need to proceed according to the Quran. …”	Interviewee #19: “I asked a sheikh about my situation… You know what he said? He said that everything is in God’s hands, and we can’t fight God’s decision. He said: ‘I personally don’t know what to tell you, whether you should abort or not – whether it is allowed or not, because it is not mentioned in the Quran. Forbidden or permitted in your case… I cannot sanction it… because it is killing a living soul. But! It’s up to you, whether you can bear the mourning and sadness that come after you have a child like this.’ I felt like he was hinting that I should abort but that he could not officially permit it as a man of religion. That’s why now I don’t believe sheikhs, their opinions and feelings. Whatever decision they make I think is based on religious uncertainty because these things are not explained clearly in our religion.”	Influence of Religious Leaders
Interviewee #20: “I know about two cases in which they said one thing, but another thing happened. Like when they said the child would be born handicapped but he was born completely healthy. But this still didn’t make me feel good.”	Interviewee #4: “My neighbor told me that she was also told that her child would be disfigured, that she would be born with a disfigured face. But she was born healthy and beautiful. When you are in this situation, you need something to hope for.”	Trust/Mistrust
Interviewee #5: “Not all of the tests are accurate. I have heard lots of stories about babies whose doctors said that they would be born with serious defects but they were born completely healthy. I still think that the degree of accuracy is 50%.”	Interviewee #2: “In my opinion, genetic tests are important… but only if the results are correct.”	Perceptions/Attitudes towards Genetic Testing

### Religious influence

All of interviewees mentioned the belief in God as a reason to continue the pregnancy despite the test results. Along with the religious prohibition to terminate pregnancy, half of the interviewees emphasized fatalism as a reason to continue the pregnancy. The women mentioned fatalistic beliefs that helped maintain optimism regarding the pregnancy, even if they chose to undergo all of the recommended medical tests.

### Religious and social factors, without reference to emotional component according to doctors

The study findings of the doctors indicated that the decision-making process for women regarding pregnancy termination or continuation was affected by religious, cultural, social and personal considerations. The doctors did not mention “maternal affect” as a factor for Muslim women in their decision to continue their pregnancies.

### Maternal affect

In all of the interviews with the women, they mentioned the emotional element as dominant in decision-making. All of the interviewees discussed bonding with the fetus: “I will not give him up,” “I feel the fetus moving,” “my flesh and blood.” This was surprising because it was raised in all of the interviews, along with the religious element, and was stronger than normative and cultural reasons (Table 4).

**Table 4:** Selected quotes from women participants in the study indicating “maternal emotion” as the main reason for refusing an abortion

Age (years)	Number of pregnancy	Quotes
31	3	“I’m the only one responsible for taking care of her. I’m a mother! I don’t care about anything except my daughter. I won’t give her up by any means. I will never give up my daughter.”
35	3	“Am I making the right decision? Am I thinking only about myself? I don't know. But I am also saying that I will not give up my son. He’s my son. He’s moving in my womb. I can’t… I won’t give him up.”
21	1	“What encouraged me is that this is my daughter. She is part of me. My flesh. I am so sorry for every tear I shed during the pregnancy. I swear to you. Except for the special love I give her… My heart saw her before my eyes. That’s why I felt her before seeing her. She holds my heart. I am very proud of her.”
37	4	“Human, let’s say maternal emotion. I can’t kill a soul … I couldn’t. I don’t have the strength to do that. She is my daughter. I feel her deep in my heart. I can’t even imagine giving her up.”
25	2	“I’m a mother. And to be the mother of another son… That’s what encourages me to stand by my decision to bring my second son into the world. I don’t need another reason to explain to you why I refuse to abort him. He is moving inside me. I can feel him. How could I give him up? When he’s hungry he knocks on my stomach. When I eat something sweet he thanks me. I feel his thank you knocks and can tell the difference between them and his hunger knocks.”
32	4	“No matter what the child’s condition is… When I felt the fetus’s movements I believed everything was fine and that encouraged me. I became attached to him. My heart saw him and felt him. I could not think of giving him up and aborting for one moment.”
27	2	“I expected that it would be hard for him… because I already knew what was going to happen. Because I experienced it before him with his third sister. I know that giving birth to such a child will require a lot of treatment and hospitalization. Still I won’t give him up. He’s my son. He is part of me.”
32	1	“When I found out I was pregnant my life filled with joy. Happiness. From the day I got married I dreamed of becoming a mother. My dream came true. I will never give up my son. I don’t know. I have an internal maternal feeling there’s nothing wrong with my fetus. I can feel it.”

### Effective risk communication according to the doctors: staying objective

All of the doctors emphasized their obligation to respect the woman’s decision and most described maintaining a “neutral stance” indicative of the approach of nondirectiveness that helps patients to make the best decisions for themselves and their families as judged from their own perspectives [38]. As part of maintaining neutrality, according to all interviewees, most of the doctors emphasized providing the patient with all relevant information without making a personal recommendation.

“My duty is only to explain to her. I am not responsible for telling her whether to continue or terminate the pregnancy…”(Interviewee 2) Most doctors did not address the emotional component of risk communication at all. However, three of them did mention the emotional component, only to contend that as doctors, it is not their job to offer emotional support. The following quote shows that the interviewee believes that the women need emotional support, but that doctors do not need to be the ones providing this support:

“Of course you have to support the woman emotionally, you can involve a psychologist, social worker but from my experience, women usually tolerate it well.”(Interviewee 5)

Only one addressed the emotional aspect of communicating the risk and mentioned women’s discourse about guilt: “They start asking, could it be because I did this or because I didn’t do that, some of them develop guilty feelings, I have to explain to them that it’s not their fault…” (Interviewee 4)

According to all of the doctors interviewed, providing information not only enables the women make decisions but also helps them cope with the diagnosis. An interesting point that arose repeatedly in the interviews with medical specialists regarding the information conveyed to the patient concerned the legal question of medical malpractice. It appears that doctors’ fear of malpractice suits is a central factor affecting the importance they attached to conveying complete information. One interviewee explained that the fear of malpractice suits influences the relationship between doctor and patient:

“We present the results to the family and the family or couple are the ones who decide….but people do not always think this through, and therefore they don’t make the right decision, and sure enough… After the baby is born with a defect they remember ‘the doctor did not explain it to us, he did explain it to us,’ … Today doctors seek to protect themselves against lawsuits…” (Interviewee 3)

### Effective risk communication: Doctors’ emotional involvement

The patient interviews indicate that how the attending doctor communicated the risk influenced the women’s decisions. One interviewee described her feelings regarding how the doctor treated her:

“The tests done during pregnancy are very important. But you need a doctor who knows how to proceed and how to make decisions. A doctor who knows how to communicate and to convey the message to women in a way that doesn’t insult them. Doctors today learn about women… every new incident teaches them something new – as though we were mice in a lab experiment… They don’t care about our feelings. They treat us like animals without feelings or hearts.”

Doctors’ lack of empathy and dry communication of risk without addressing the emotional aspect was difficult for most of the women. Moreover, some interviewees maintained that the doctors pressured them to terminate the pregnancy, without considering their view:

“I’m mad about how the doctor talked to me. There are doctors who don’t know how to talk or communicate with women. As if they’re talking to a lump of clay or piece of a wall that does not have a heart or feelings. He just told me about the diagnosis in a very rough way. He said it like this: ‘Get off the bed, the fetus in your womb is deformed… you have to abort it… Come on, Abdallah, sign for an abortion.’ I was shocked.”

Some of the interviewees also mentioned that they felt that the doctors were afraid of malpractice suits and therefore encouraged pregnancy termination. Some of the interviewees also expressed distrust of the doctor and of the accuracy of the tests: “…Not every medical person can decipher the results correctly. I think the level of accuracy is 50% or I would even say less than 50%.”

## Discussion

This study contributes both on the theoretical and applied levels in light of a substantial rise in congenital anomalies among the Arab Muslim community in Israel [58]. The factors that affect the decisions of Muslim women in Israel whether to abort or to continue their pregnancy after discovering a congenital anomaly are diverse and involve cultural and social aspects (especially family), but mainly religious considerations. Most studies about Muslim women’s decision to continue their pregnancies despite test results that indicate congenital anomalies reveal that the factors that influence their decision are religious, socioeconomic, and cultural considerations and a worldview influenced by a belief in fatalism [59–62]. Many of our findings conform with these known factors.

The finding that was unexpected and that recurred in the interviews with the women was the emotional element. The women mentioned that they felt the fetus’s movements and that giving it up would be giving up motherhood. Even studies that raise other considerations (sex of the fetus, fetal age, quality of life of the newborn) do not raise the emotional element [63, 64]. This has not been raised in previous studies of Muslim women at high risk of giving birth to a fetus with a defect, and that is a significant contribution of the present study.

According to risk communication literature, emotional affect is important [39–41]. These studies reinforce the findings of this study, that for women, decision-making is affected not only by information but also by their feelings towards the fetus and towards the doctor. Here it should be emphasized that emotion does not occur in a vacuum and is often influenced by cultural norms, pressures and considerations. Decisions are influenced by many factors, some of which are deeply embedded in society and include social status, geographical location, level of social, cultural and economic development, as well as access to jobs and patriarchal norms [65]. In addition, it has been noted that the collectivist tendency is more widespread and deeply rooted than individualist tendencies in Arab societies, and as such, individual decisions can be seen as stemming from the values of the collective [66].

However, some relatively recent studies discuss emotions as stemming from individual considerations, and not influenced by the dominant cultural practices in a totalizing way [67]. Emotions can be influenced by external factors, but at the same time, can be seen as autonomous feelings that the women interviewees feel towards the fetus they are carrying. Studies indicate that the process of testing and deciding to abort are emotionally complex. Many studies have suggested a correlation between doctor-patient communication and patient compliance with the treatment suggested by the doctor [68]. Care communication literature and the patient-centered communication approach emphasize the importance of the emotional element on patient health [69]. Productive dialogue and communication must include technical and emotional considerations [44].

**Fig. 1:**
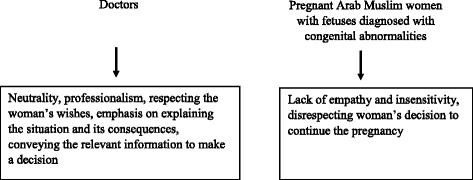
Perception of professional distance in conveying information from doctor to patient

The findings of this study emphasize the disparities between the doctors and the women regarding the emotional element in their communication. The doctors in this study maintained it was important for them to respect the patient’s decisions and to refrain from emotional involvement (non-directive counselling). Many of the women interviewees felt that this expressed insensitivity. The interviews with the women found that many of them felt the doctors were pressuring them to terminate the pregnancy. Therefore, the doctors’ request to repeat the medical diagnosis, interpret it, explain its significance and consequences – which the doctors perceived as professional behavior – was sometimes interpreted by the women as intervention and disrespect for the woman’s decision to continue the pregnancy. In that respect there is a gap between the doctors’ perception of themselves as neutral, professional and respectful of the patient’s decision, and the patients’ perception of the doctor’s behavior as insensitive. This can be described by a risk communication paradigm (Fig. 1).

Furthermore, the doctors are perceived as bearers of bad news [70]. Many doctors choose to maintain emotional distance from patients to avoid angry or critical reactions [71]. Doctors perceive themselves as objective professionals, who should not express emotions when conveying bad news to a patient [72]. For patients, it is important for the doctor to break the news with direct and concise explanations, including her in decision-making, treatment and prognosis, encouraging the patient to express feelings and questions, and adjusting the message to the patient’s culture, religion and language [73]. Not only does communication with patients impact on the patient’s understanding of the risks, compliance with the suggested treatment and the outcome of the treatment itself, but studies have found that such communication can also prevent lawsuits [45]. It is important for doctors to understand the emotional element in risk communication, both in respecting women’s emotions and in creating an emotional interaction.

## Conclusions

The present study shows that the emotional element is no less dominant than religious and social elements. The emotional bond the women create with the fetus during their pregnancies, and the absence of doctors’ empathy when communicating risk impact women’s decision-making. Doctors should undergo training in risk communication, to emotionally support women in the decision-making process. Doctors’ difficulty dealing with the emotional aspects of communicating bad news suggests the need for cooperation between doctors and social workers in communicating with patients. Based on the findings of this study, we recommend compiling guidelines for doctors to help them to show empathy and to improve communication with Arab Muslim patients. Based on our findings, it seems that doctors approach the women from a stereotypcal perspective that assumes that the religious aspect will be the decisive one in making the decision to continue or terminate the pregnancy. In addition, our findings show that doctors do not think it is their job to address the emotional component, but we want to suggest that this is a crucial aspect of their jobs. They need to address the emotional element in their communication with patients or arrange for a social worker to accompany them in their meetings with patientsto offer emotional support to the female patients who face a difficult decision. We recommend for further studies on how the emotional element can be developed in the doctors’ communication with patients pregnant with fetuses diagnosed with congenital anomalies.

### Study limitations

Limitations of the present study include that since it is a qualitative study it does not include a representative sample of the study population. Another limitation is that the research is based only on self-reporting and we did not carry out observations of the patient-doctor interaction. Limitations include the recruitment from one area in one country and snowball sampling method. We asked doctors their views on pregnancy termination, but we did not ask them about their religious affiliation or whether or not their views were influenced by religious beliefs.

## Abbreviation

OB/GYN: Obstetrics and Gynecology
